# Comprehensive and integrative analysis identifies microRNA-106 as a novel non-invasive biomarker for detection of gastric cancer

**DOI:** 10.1186/s12967-018-1510-y

**Published:** 2018-05-15

**Authors:** Qiliang Peng, Yi Shen, Kaisu Lin, Li Zou, Yuntian Shen, Yaqun Zhu

**Affiliations:** 1 Department of Radiotherapy & Oncology, The Second Affiliated Hospital of Soochow University, San Xiang Road No. 1055, Suzhou, 215004 Jiangsu China; 2 Institute of Radiotherapy & Oncology, Soochow University, Suzhou, China; 3 Suzhou Key Laboratory for Radiation Oncology, Suzhou, China; 4 Department of Oncology, The Second Affiliated Hospital of Soochow University, Suzhou, China; 5 Department of Oncology, Nantong Rich Hospital, Nantong, China

**Keywords:** Gastric cancer, Meta-analysis, System biological analysis, Diagnosis

## Abstract

**Background:**

Recently, accumulating evidences have revealed that microRNA-106 (miR-106) may serve as a non-invasive and cost-effective biomarker in gastric cancer (GC) detection. However, inconsistent results have prevented its application to clinical practice.

**Methods:**

As a result of this, a comprehensive meta-analysis was conducted to evaluate the diagnostic performance of miR-106 alone and miR-106-related combination markers for GC detection. Meanwhile, an integrative bioinformatics analysis was performed to explore the function of miR-106 at the systems biology level.

**Results:**

The results in our work showed that sensitivity of 0.71 (95% CI 0.65–0.76) and specificity of 0.82 (0.72–0.88), with the under area AUC (area under the curve) value of 0.80 (0.76–0.83) for miR-106 alone. Prospectively, miR-106-related combination markers improved the combined sensitivity, specificity and AUC, describing the discriminatory ability of 0.78 (0.65–0.87), 0.83 (0.77–0.89) and 0.88 (0.85–0.90) in the present analysis. Furthermore, targets of miR-106 were obtained and enriched by gene ontology and Kyoto Encyclopedia of Genes and Genomes pathway analysis, revealing their associations with the occurrence and development of GC. Hub genes and significant modules were identified from the protein–protein interaction networks constructed by miR-106 targets and found closely associated with the initiation and progression of GC again.

**Conclusions:**

Our comprehensive and integrative analysis revealed that miR-106 may be suitable as a diagnostic biomarker for GC while microRNA combination biomarkers may provide a new alternative for clinical application. However, it is necessary to conduct large-scale population-based studies and biological experiments to further investigate the diagnostic value of miR-106.

## Background

Gastric cancer (GC), a major public health challenge, is one of the leading causes of cancer death worldwide [1]. There are limited detection methods for early diagnosis and few effective screening procedures in some countries. The most reliable program for diagnosis is mainly based on endoscopy and biopsy [2]. However, this is invasive and inconvenient for patients to undergo. Consequently, most patients can only be diagnosed precisely in advanced stages when the clinical outcomes are poor [3]. Therefore, there is a great need to explore new accurate and efficient, preferentially non-invasive, markers for early detection of GC.

In recent time, accumulating evidences have suggested that microRNAs may serve as novel biomarkers for cancer detection. MicroRNAs are a class of small non-coding RNAs with intermediate posttranscriptional regulation of the target genes [4]. A large number of studies have demonstrated that microRNAs play vital roles in a wide variety of physiological processes including cancer cell growth, differentiation, invasion, and metastasis [5]. Moreover, a number of studies have indicated that circulating microRNAs have high degree of stability and tolerance even under unfavorable physiochemical conditions including extreme variations in pH, temperature and freeze–thaw cycles [6]. It is also promising that microRNAs have outstanding stability in multiple clinical samples including plasma, serum, feces and tissue, which enables them to be detectable effortlessly [7]. Given their critical involvement in the vital biological processes and perfect biomarker features mentioned above, microRNAs could be considered as good candidates for using as non-invasive markers, and the application of them as biomarkers for early detecting GC is viable [8].

As one of the most representative microRNA biomarkers, microRNA-106 (miR-106) has been extensively studied by a great number of researches in several cancers. MiR-106 belongs to the miR-17 family, one of the most common studied onco-microRNA groups, which includes miRs-17, -20a, -20b, -93, -106a and -106b. MiR-106a is a member of the miR-106a-92 cluster located on chromosome Xq26.2 while miR-106b is located at 7q21 [9, 10]. There have been several studies indicating that both of investigated miR-106 could be expressed in the same individuals of gastric tumour tissues. Several studies have previously reported that circulating miR-106 could specifically serve as a pivotal and promising biomarker for GC [11]. Nevertheless, the suitability of circulating miR-106 in early detection and diagnosis of GC remains inconsistent due to different sample sizes, disease statuses, sample sources, detection methods, and other uncontrolled factors. Moreover, the potential molecular mechanism of miR-106 is still poorly understood for the present insufficient knowledge.

In the present study, a comprehensive meta-analysis was performed to obtain a better understanding of the clinical feasibility of miR-106 as promising biomarker for early detection and diagnosis of GC. By focusing not only on a single miR-106 marker, we explored whether combination biomarkers based on miR-106 are more effective than individual miR-106. Furthermore, an integrative bioinformatics analysis was carried out to evaluate the functions of miR-106 at the systems biology level.

## Methods

### Literature search strategy

A comprehensive computerized literature search for articles (up to December 27, 2017) was carried out based on several electronic databases including PubMed, EMbase, Web of Science and the Cochrane Library using the following search terms: (“cancer” OR “tumor’’ OR “carcinoma” OR “neoplasm”) AND (“gastric” OR “stomach’’ OR “gastrointestinal’’ OR “digestive” OR “GC”) AND (“microRNA-106” OR “miR-106” OR “miR-106a” OR “miR-106b”). In addition, the references of identified articles were examined for all relevant studies.

### Eligibility criteria

The studies qualified to be included should meet the following criteria: (1) they investigated the potential of circulating (blood, serum, and plasma) or other sources of miR-106 for detecting GC; (2) they used the gold standard to make a definitive diagnosis of GC; (3) they provided adequate data which can be used to calculate the rates of true positive (TP), false positive (FP), false negative (FN), and true negative (TN).

In addition, the studies were excluded if (1) they were obviously not associated with our topic; (2) they published in forms of reviews, letters, case reports, or editorials; (3) they were non-English publications; or (4) they provided unqualified data.

### Data extraction

Data were collected independently by two investigators (Peng and Shen) from the articles based on standardized forms. The following characteristics from each study were extracted: (1) first author; (2) publication year; (3) research country; (4) study population; (5) patient characteristics (age, gender, cancer type, etc.); (6) sample sources; (7) participants numbers; (8) miR-106 measuring methods, (9) diagnostic data including sensitivity, specificity, true positive (TP), false positive (FP), false negative (FN) and true negative (TN).

### Quality assessment

The quality of each study enrolled in our analysis was assessed independently by two investigators on the basis of the QUADAS-2 (Quality Assessment of Diagnostic Accuracy Studies 2) [12]. The QUADAS-2 assessment tool contains 4 domains including patient selection, index test, reference standard, and flow and timing supported by signaling questions (yes, no, or unclear), risk of bias (high, low, or unclear) and concerns about applicability (high, low, or unclear). An answer of “yes” gets a score of 1, which indicates a low risk of bias, while an answer of “no” or “unclear” gets a score of 0 which means a potential high risk of bias.

### Statistical analysis for meta-analysis

The overall diagnostic results were estimated using the TP, FP, FN, and TN test results extracted directly from each study or via recalculation based on sensitivity and specificity together with other data retrieved from each study. The bivariate meta-analysis model was applied to calculate the pooled sensitivity, specificity, positive likelihood ratios (PLR), negative likelihood ratios (NLR), and diagnostic odds ratio (DOR) along with their corresponding 95% confidence intervals (CIs) [13]. The summary receiver operator characteristic (SROC) curve was generated based on the sensitivity and specificity of each study [14]. In addition, we calculated the corresponding area under the SROC curve (AUC) for the quantitative assessment of diagnostic power. Furthermore, the heterogeneity across studies was examined by using Q test and I^2^ test [15]. A P value ≤ 0.1 from Q test and an I^2^ ≥ 50% from I^2^ test suggest the presence of significant heterogeneity among eligible study. The heterogeneity caused by threshold effect was quantified using Spearman correlation analysis. Possible sources of heterogeneity in the aspect of non-threshold effect were explored by carrying out subgroup, meta-regression, and sensitivity analyses [16]. We examined the potential publication bias through Deeks’ funnel analysis [17]. Statistical analyses were performed in STATA (version 14.0) and Meta-DiSc statistical software (version 1.4) software. Values of P < 0.05 were considered to represent statistical significance.

### Integrative functional analysis of miR-106

To further explore the function of miR-106, an integrative functional analysis was performed. Targets of miR-106 were obtained from miRTarBase, a resource for experimentally validated microRNA-target interactions [18]. Updated in 2018 with plenty of integrative improvements, miRTarBase has been a more comprehensively annotated microRNA-target interactions database approved by experimental evidence compared with its previous versions and other databases in the field of microRNA-target prediction research. We conducted GO and KEGG pathway enrichment analysis by separately mapping the target mRNAs of miR-106a and miR-106b to the online tool DAVID (Database for Annotation, Visualization, and Integrated Discovery) [19–21]. P-value < 0.05 and count ≥ 2 were considered as the cut-off criteria.

### PPI network analysis

The target mRNAs regulated by miR-106a and miR-106b were mapped to the Search Tool for the Retrieval of Interacting Genes (STRING) database to retrieve the PPI information, respectively [22]. In the present study, we only selected the PPI data with the combined score > 0.4. Network visualization was used the powerful tool Cytoscape [23]. Meanwhile, we identified several hub genes of miR-106a and miR-106b targets by using three different methods including betweenness centrality, closeness centrality and degree centrality based on the plug-in CytoNCA [24]. In addition, the significant modules in the PPI network were screened by plug-in Molecular Complex Detection (MCODE) of Cytoscape. As for a further comment, KEGG pathway analysis was performed with the hub genes and genes involved in the selected modules by DAVID.

## Results

### Study selection

**Fig. 1:**
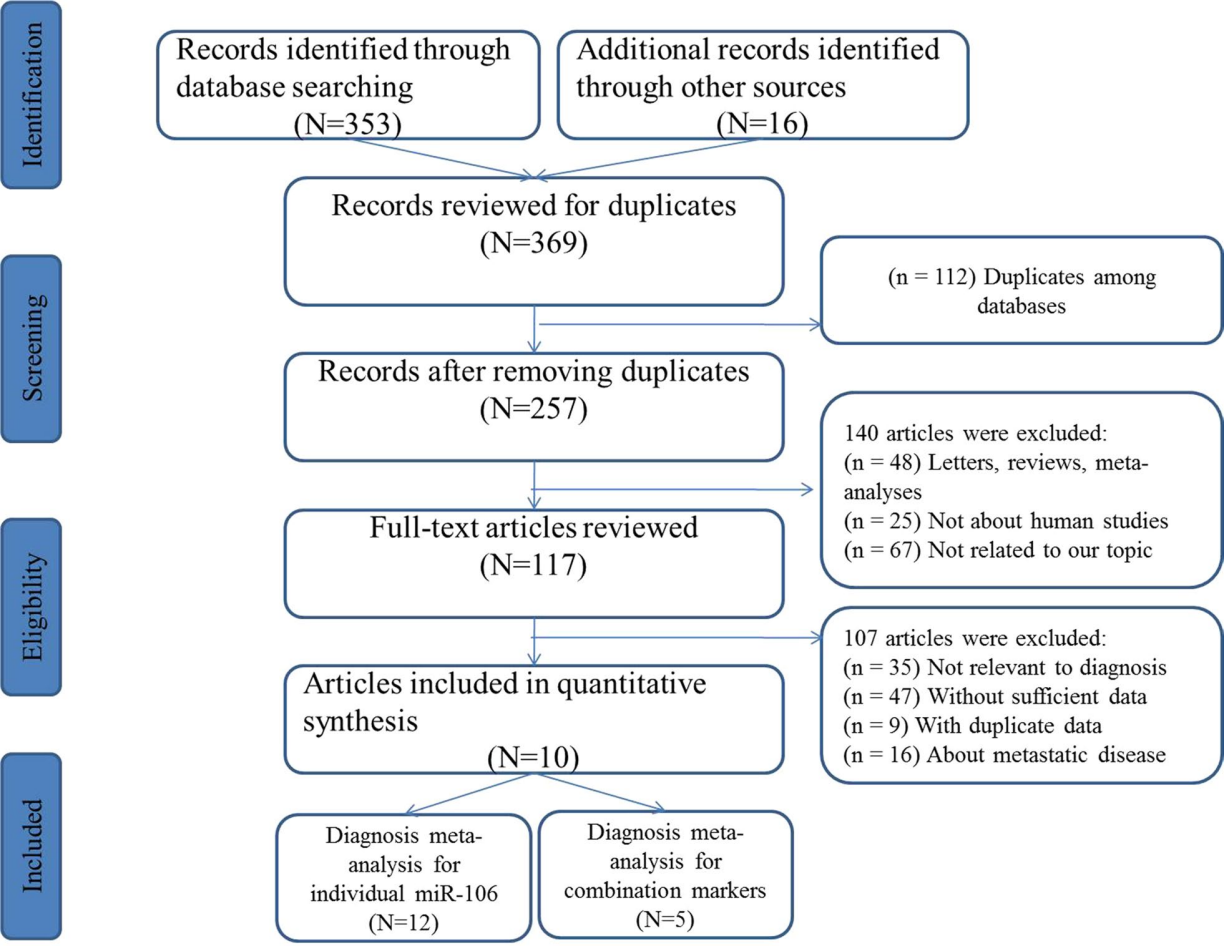
Flow diagram of the study selection process

After initial searches of the databases mentioned above, a total of 369 articles were retrieved preliminarily, and the flow diagram of the literature search was shown in Fig. 1. After careful exclusion of inappropriate ones according to the inclusion criteria step by step, ten published articles were finally included in our study, including twelve studies for miR-106 alone and five for miR-106-related combination markers [25–34].

### Baseline characteristics of included studies

Among the included publications for evaluating miR-106 alone in detecting GC, a total of 10 articles on 12 studies were analyzed involving 788 cases and 701 healthy people as the control group. There were five studies investigating serum miR-106, five studies assessing plasma miR-106, one study evaluating tissue miR-106 and one study involving miR-106 in gastric juice. Among all these studies, six focused on miR-106a and while 6 studies concentrated on miR-106b. None of the studies in Table 1 evaluated both miRNAs (miR-106a and miR-106b). With respect to value of miR-106-related combination markers, 4 publications on 5 studies involved 322 patients and 263 controls. Sample sources were all serum (n = 5). All the studies measured the expression level of miR-106 and the related combination markers by using the quantitative real-time polymerase chain reaction (qRT-PCR) assay. The detailed information was shown in Tables 1 and 2. The quality assessments of the included studies indicated that overall they were of moderate to high quality, which enhanced the reliability of our analysis.

**Table 1:** The main features of the included studies on individual miR-106

First author	Year	Country	Ethnicity	Sample size (case/control)	N	Male/femal (case)	Sample source	Methods	MicroRNA	AUC	Sencitivity (%)	Specificity (%)	QUADAS
Tsujiura et al.	2010	Japan	Asian	69/30	99	NA	Plasma	qRT-PCR	miR-106b	0.721	80.00	63.00	4
Zhou et al.	2010	China	Asian	90/27	117	63/27	Serum	qRT-PCR	miR-106a	0.684	48.15	90.24	5
Cai et al.	2013	China	Asian	90/90	180	66/24	Plasma	qRT-PCR	miR-106b	0.773	66.00	80.00	5
Cui et al.	2013	China	Asian	42/99	141	32/10	Gastric juice	qRT-PCR	miR-106a	0.871	73.80	89.30	5
Shiotani et al.	2013	Japan	Asian	64/64	128	41/23	Serum	qRT-PCR	miR-106b	0.610	55.60	70.30	6
Shiotani et al.	2013	Japan	Asian	62/70	132	47/15	Serum	qRT-PCR	miR-106b	0.700	75.80	51.40	6
Zeng et al.	2014	China	Asian	40/36	76	28/12	Serum	qRT-PCR	miR-106b	0.856	75.00	92.50	5
Hou et al.	2015	China	Asian	80/60	140	42/38	Plasma	qRT-PCR	miR-106a	0.895	77.50	93.80	4
Wang et al.	2017	China	Asian	110/110	220	NA	Serum	qRT-PCR	miR-106a	0.786	62.90	88.5	5
Li et al.	2017	China	Asian	65/65	130	50/15	Plasma	qRT-PCR	miR-106b	0.898	86.20	92.30	5
Yuan et al.	2017	China	Asian	28/28	56	22/6	Tissue	qRT-PCR	miR-106a	0.666	62.50	63.60	6
Yuan et al.	2017	China	Asian	48/22	70	38/10	Plasma	qRT-PCR	miR-106a	0.828	77.10	63.60	6

*N* number of participants, *NA* not available, *AUC* area under the curve, *QUADAS* quality assessment of diagnostic accuracy studies

**Table 2:** The main features of the included studies on miR-106-related combination markers

First author	Year	Country	Ethnicity	Sample size (case/control)	N	Male/femal (case)	Sample source	Methods	MicroRNA	AUC	Sencitivity (%)	Specificity (%)	QUADAS
Zhou et al.	2010	China	Asian	90/27	117	63/27	Serum	qRT-PCR	miR-106a and miR-17	0.741	62.96	80.49	5
Shiotani et al.	2013	Japan	Asian	62/70	132	47/15	Serum	qRT-PCR	miR-106b and miR-21	NA	69.00	69.40	6
Zeng et al.	2014	China	Asian	40/36	76	28/12	Serum	qRT-PCR	miR-106b and miR-17	0.913	83.30	87.50	5
Wang et al.	2017	China	Asian	110/110	220	NA	Serum	qRT-PCR	miR-106a and miR-19b	0.814	71.08	82.44	5
Wang et al.	2017	China	Asian	20/20	40	NA	Serum	qRT-PCR	miR-106a and miR-19b	NA	95.00	90.00	5

*N* number of participants, *NA* not available, *AUC* area under the curve, *QUADAS* quality assessment of diagnostic accuracy studies

### Diagnostic value of miR-106 in GC

**Fig. 2:**
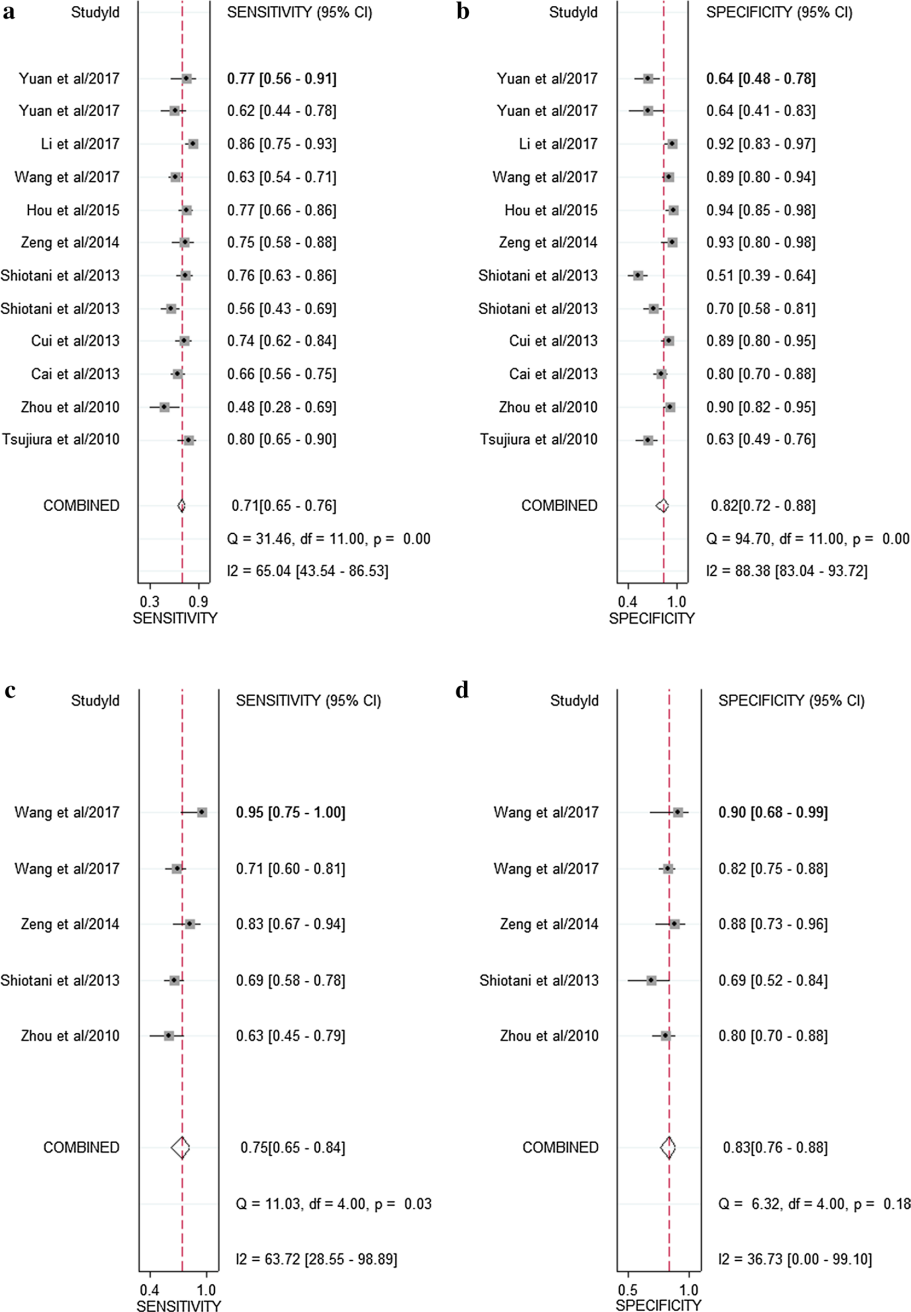
Forest plots of sensitivities and specificities from test accuracy studies in the diagnosis of GC. **a**, **b** Forest plots of sensitivities and specificities for miR-106 alone; **c**, **d** forest plots of sensitivities and specificities for miR-106-related combination markers

**Fig. 3:**
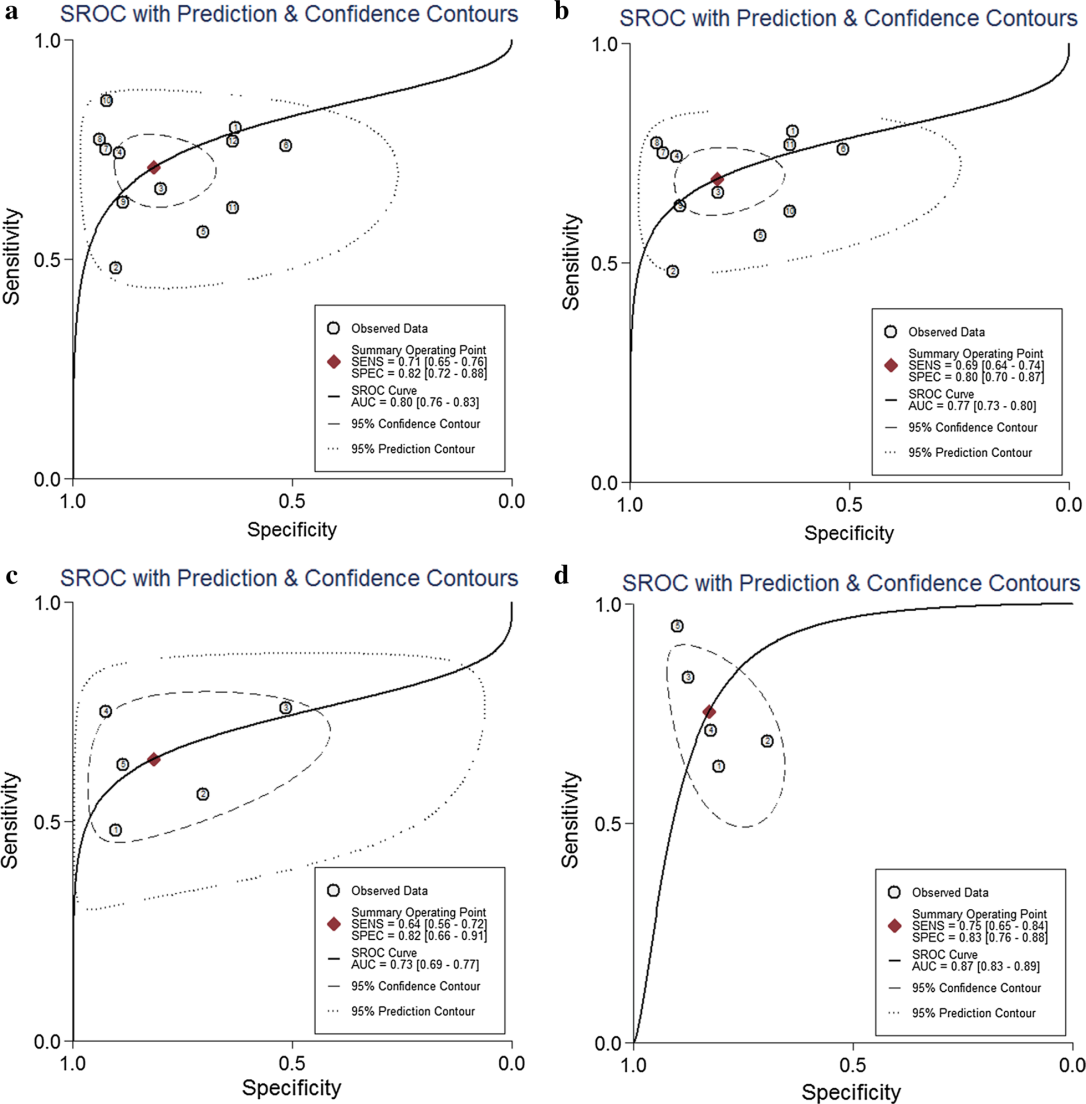
The SROC curves in the diagnosis of GC. **a** SROC curve of overall including the outliers for miR-106 alone; **b** SROC curve of outliers excluded for miR-106 alone; **c** SROC curve for miR-106 alone in serum samples; **d** SROC curve for miR-106-related combination markers in serum samples. *SROC* summary receiver operator characteristic, *GC* gastric cancer

As indicated in the forest plot (Fig. 2a, b), the existence of significant heterogeneity among individual studies was observed as the Q value was 31.46 (P < 0.001) and I^2^ value was 65.04% (95% CI 43.54–86.53) for sensitivity, and the Q value was 94.70 (P < 0.001) and I^2^ value was 88.38% (95% CI 83.04–93.72) for specificity. Thus, a random-effects model was applied to evaluate the pooled results. Overall, the pooled assessment outcomes were as follows: sensitivity, 0.71 (95% CI 0.65–0.76); specificity, 0.82 (0.72–0.88); PLR, 3.86 (2.48–6.01); NLR, 0.36 (0.28–0.45); and DOR, 10.84 (5.88–20.00), respectively. The pooled PLR meant that patients with GC had a nearly four-fold greater chance of being miR-106 positive than that for patients without GC while the combined NLR of 0.36 indicated that expected proportion of patients having GC is 36% if the miR-106 is negative. The DOR value suggested that someone who was screened to be positive for GC with a high expression of miR-106 had a 10.84-fold higher possibility of actually suffering from GC than someone with a negative GC result. Finally, the SROC curve (Fig. 3a) was plotted and the corresponding AUC was calculated of 0.80 (0.76–0.83), indicating moderate diagnostic accuracy overall.

The threshold effect may result from the differences between sensitivity and specificity. The representative way of assessing the threshold effect is estimating Spearman’s correlation coefficient of sensitivity and specificity. According to our results, Spearman’s correlation coefficient was calculated to be − 0.049, with a P value of 0.88 (P > 0.05), suggesting that no obvious heterogeneity generating from the threshold effect.

In the present study, subgroup analysis was applied in order to identify the potential sources of heterogeneity (see Table 3). The results implied that plasma miR-106 had relatively high diagnostic accuracy for GC detection compared with serum miR-106, with sensitivity of 0.77 (95% CI 0.69–0.83) vs. 0.64 (0.56–0.72), specificity of 0.82 (0.67–0.91) vs. 0.82 (0.66–0.91), and AUC of 0.83 (0.80–0.86) vs. 0.73 (0.69–0.77). Among the twelve studies, ten studies detected the miR-106 in circulating samples. Hence, subgroup analysis was also performed by circulating samples. The pooled sensitivity, specificity and AUC for circulating miR-106 were 0.71 (0.64–0.78), 0.82 (0.72–0.89), and 0.81 (0.77–0.84). We found no significant difference in the diagnostic accuracy between studies with miR-106a and miR-106b, with AUC of 0.78 (95% CI 0.74–0.82) vs. 0.81 (0.77–0.84) although miR-106b exhibited higher diagnostic sensitivity of 0.74 (0.65–0.81) compared with studies with miR-106a, for which the value was 0.68 (0.60–0.75) while miR-106a was associated with specificity of 0.85 (0.74–0.92) compared with the value for miR-106b of 0.78 (0.63–0.88). Subgroup analysis by the sample size suggested that a large sample size had higher specificity and AUC of 0.85 (0.75–0.91) and 0.82 (0.78–0.85) compared to a small sample size with specificity of 0.73 (0.56–0.85) and AUC of 0.75 (0.71–0.78), indicating large sample size had relatively overall high diagnostic accuracy than small sample size, although a small sample size exhibited higher sensitivity of 0.74 (0.66–0.80) compared with a large sample size of 0.70 (0.62–0.77).

**Table 3:** Pooled results of diagnostic accuracy of miR-106 and combination biomarkers in gastric cancer

	Analysis	Number of studies	Se (95% CI)	Sp (95% CI)	AUC (95% CI)
Individual	Country				
	China	9	0.71 (0.64–0.77)	0.87 (0.79–0.91)	0.83 (0.80–0.86)
	Japan	3	0.70 (0.62–0.76)	0.61 (0.54–0.68)	0.70 (0.67–0.74)
	Sample size				
	< 100	4	0.74 (0.66–0.80)	0.73 (0.56–0.85)	0.75 (0.71–0.78)
	> 100	8	0.70 (0.62–0.77)	0.85 (0.75–0.91)	0.82 (0.78–0.85)
	Sample type				
	Plasma	5	0.77 (0.69–0.83)	0.82 (0.67–0.91)	0.83 (0.80–0.86)
	Serum	5	0.64 (0.56–0.72)	0.82 (0.66–0.91)	0.73 (0.69–0.77)
	Circulating	10	0.71 (0.64–0.78)	0.82 (0.72–0.89)	0.81 (0.77–0.84)
	Gastric juice	1	0.74 (0.58–0.86)	0.89 (0.77–0.96)	0.87 (0.80–0.94)
	Tissue	1	0.63	0.64	0.67 (0.53–0.80)
	miRNA profiling				
	miR-106a	6	0.68 (0.60–0.75)	0.85 (0.74–0.92)	0.78 (0.74–0.82)
	miR-106b	6	0.74 (0.65–0.81)	0.78 (0.63–0.88)	0.81 (0.77–0.84)
	Overall	12	0.71 (0.65–0.76)	0.82 (0.72–0.88)	0.80 (0.76–0.83)
	Outliers excluded	11	0.69 (0.64–0.74)	0.80 (0.70–0.87)	0.77 (0.73–0.80)
Combination	Overall	5	0.78 (0.65–0.87)	0.83 (0.77–0.89)	0.88 (0.85–0.90)

*AUC* area under the curve, *Se* sensitivity, *Sp* specificity, *95% CI* 95% confidence interval

Meta-regression analysis was conducted to reveal the potential sources of the heterogeneity. We considered 5 covariates (publication year, country, sample size, sample source and miR-106 classification) may contribute to the heterogeneity. The results showed that neither publication year, nor sample size or sample source or miR-106 classification was the source of heterogeneity, but the country has influence in specificity (P < 0.05).

**Fig. 4:**
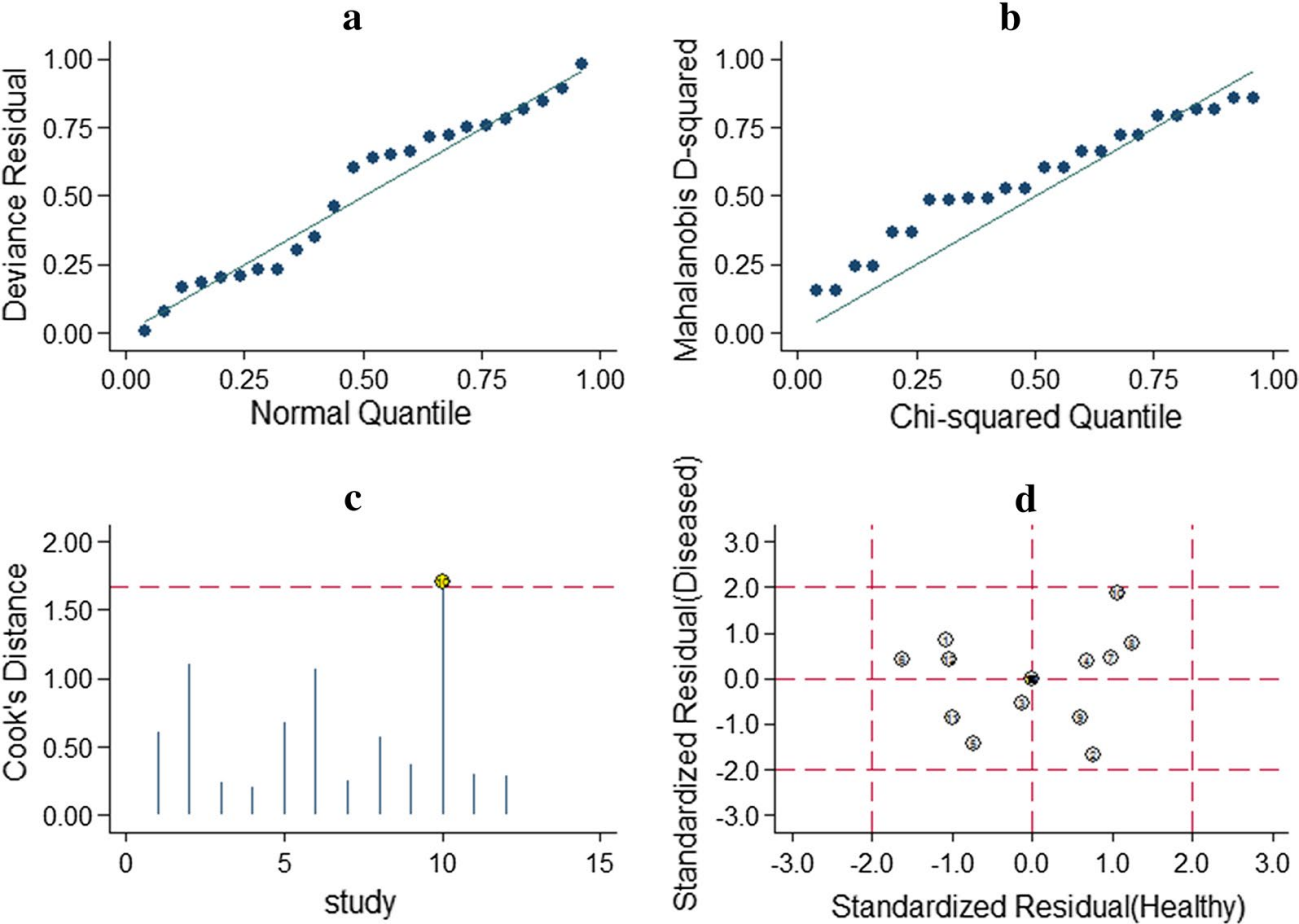
Sensitivity analysis results. **a** Goodness of fit; **b** bivariate normality; **c** influence analysis; **d** outlier detection

The goodness of fit and bivariate normality analyses suggested that the bivariate random-effects model was robust for the meta-analysis (Fig. 4). Besides that, one deviated study that may affect the robustness of the meta-analysis was identified with the method of influence analysis and outlier detection. After excluding the deviated study, no significant changes in sensitivity (0.71 vs. 0.69), specificity (0.82 vs. 0.80), PLR (3.86 vs. 3.49), NLR (0.36 vs. 0.38), DOR (10.84 vs. 9.07), and AUC (0.80 vs. 0.77) were observed between the overall analysis with and without outliers, which indicated that there was high robustness in our meta-analysis.

### Diagnostic value of miR-106-related combination marker in GC

As shown in Fig. 2c, d, I^2^ values for sensitivity and specificity were 76.97% (95% CI 56.57–97.38%, P < 0.001) and 49.95% (0.00–100.00%, P < 0.001), respectively, indicating significant heterogeneity for sensitivity and moderate heterogeneity for specificity. The pooled sensitivity, specificity, PLR, NLR, and DOR are 0.78 (0.65–0.87), 0.83 (0.77–0.89), 4.69 (2.98–7.37), 0.27 (0.15–0.46), and 17.68 (6.78–46.09), respectively. The SROC curve is shown in Fig. 3d with AUC = 0.88 (0.85–0.90), indicating a relatively higher accuracy in GC detection. Since all the combination markers were detected in serum, we compared them with serum miR-106 alone (Fig. 3c). Serum miR-106-related combination marker had a higher level of predictive power than serum miR-106 alone, with sensitivity of 0.78 (0.65–0.87) vs. 0.64 (0.56–0.72), specificity of 0.83 (0.77–0.89) vs. 0.82 (0.66–0.91), and AUC of 0.88 (0.85–0.90) vs. 0.73 (0.69–0.77).

In the present study for combination biomarkers, Spearman’s rank correlation was also evaluated to explore the potential heterogeneity from threshold effect. It was revealed from the results that heterogeneity may generate from the threshold effect, from a Spearman’s correlation coefficient of − 0.90 with P = 0.037.

### Publication bias

**Fig. 5:**
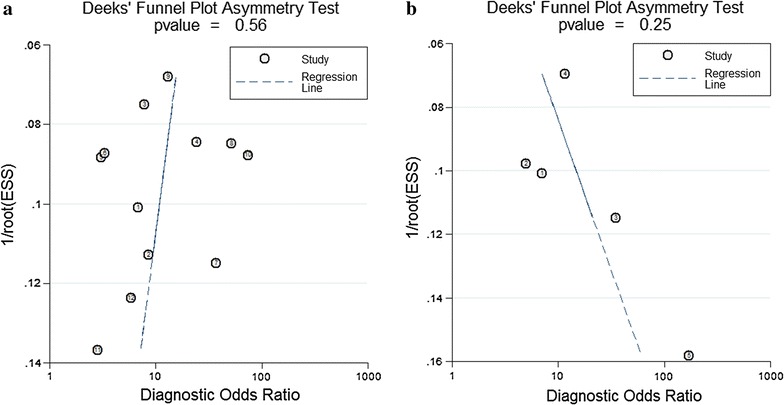
Deeks’ funnel plots for the assessment of potential bias in the meta-analysis for diagnosis. **a** Funnel plot of the studies on miR-106 alone; **b** funnel plot of the studies on miR-106-related combination markers

Begg’s funnel plot and Egger’s test were applied to evaluate the presence of publication bias (Fig. 5). The funnel plots indicated no symmetry for all enrolled studies and Deeks’ test returned P values of 0.56 and 0.45 for miR-106 and miR-106-related combination markers, respectively, revealing no obvious publication bias in the present study. However, it is difficult for judging publication bias whether or not exists due to the limited number of studies enrolled in the analysis for miR-106-related combination markers.

### Integrative functional analysis results of miR-106

Based on the above results, we wondered why miR-106 could serve as a promising biomarker for detecting GC. We supposed that if the identified miR-106 could be a biomarker of GC, the genes regulated by miR-106 should also be involved in the initiation and progression of GC. Therefore, we performed GO and pathway enrichment analyses on targets of miR-106a and miR-106b to explore the function and pathogenesis of them, respectively.

**Fig. 6:**
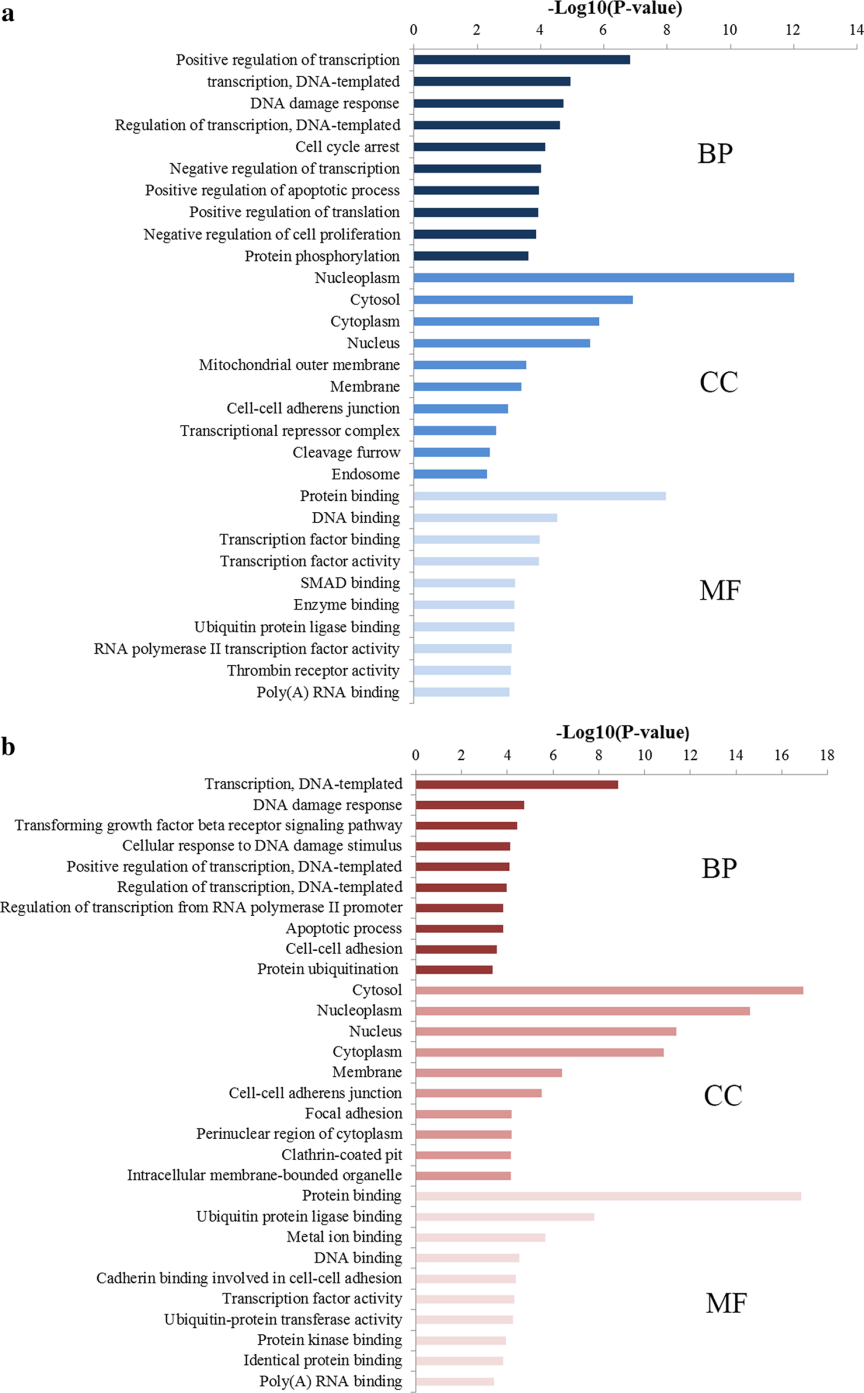
GO annotation of miR-106 target genes. **a** Top 10 GO items for target genes of miR-106a; **b** top 10 GO items for target genes of miR-106b. *GO* gene ontology, *BP* biological processes, *CC* cell component, *MF* molecular function

We performed the GO enrichment analysis by separately mapping the target genes of miR-106a and miR-106b to the online software DAVID at three different levels: molecular function (MF), cell component (CC) and biological processes (BP). The top 10 items of each GO level that were significantly enriched by the target genes were illustrated at Fig. 6. The enriched GO terms in BP for miR-106a mainly included the regulation of transcription, apoptotic process while the enriched GO terms in BP for miR-106b were also associated with regulation of transcription and apoptotic process. For the CC items, the target genes of miR-106a were enriched in the hallmarks of a cell such as nucleoplasm, cytosol, cytoplasm, nucleus and the target genes of miR-106b were also enriched in the same components including nucleoplasm, cytosol, cytoplasm and nucleus. Most GO MF items for miR-106a converged on the binding functions such as protein binding, DNA binding, transcription factor binding while most GO MF items for miR-106b were also related to protein binding, DNA binding and transcription factor binding.

**Fig. 7:**
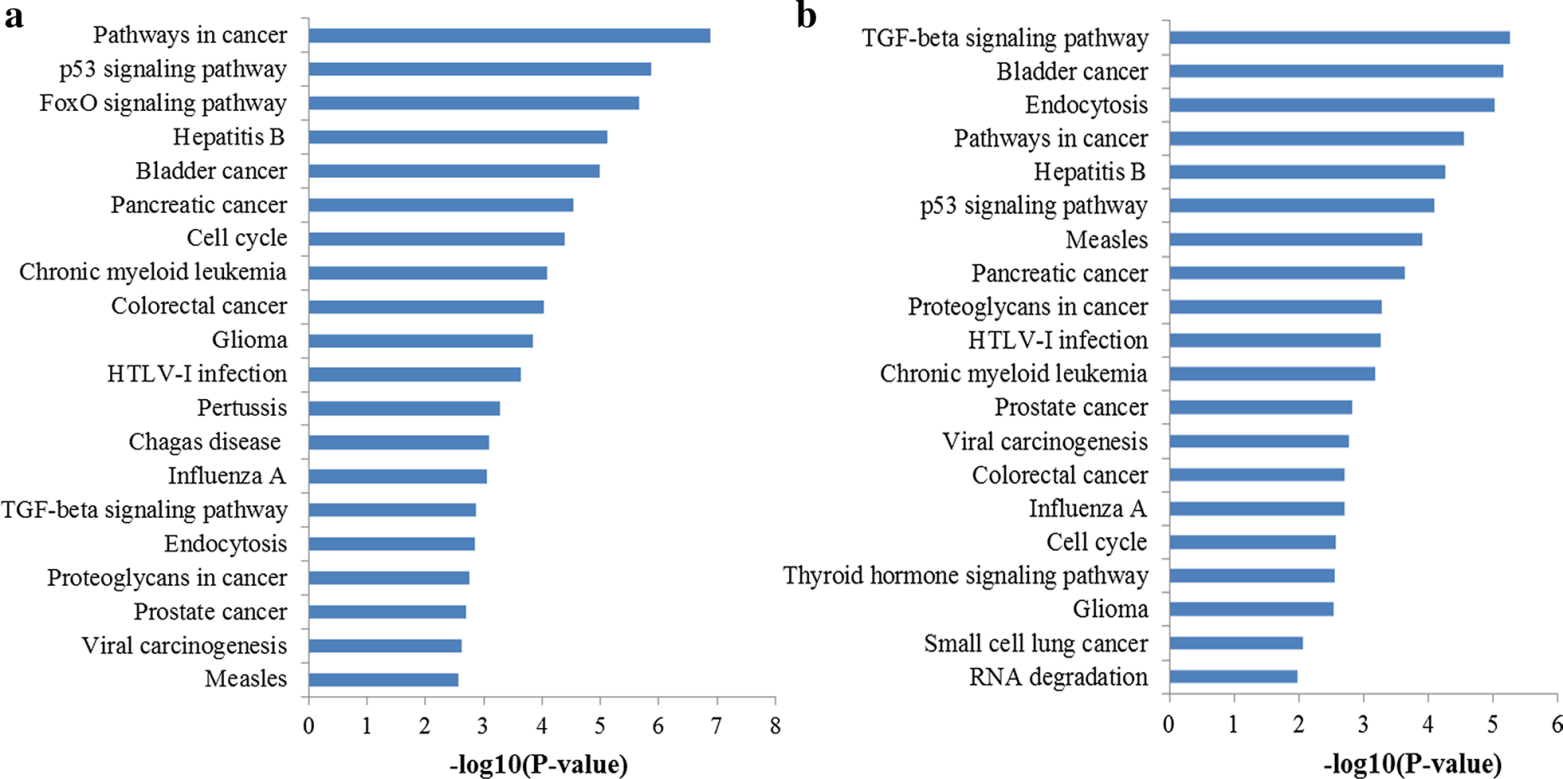
Pathway enrichment results for miR-106 target genes. **a** Top 20 pathways enriched by target genes of miR-106a; **b** top 20 pathways enriched by target genes of miR-106

The top 20 significantly enriched pathways of miR-106a and miR-106b were outlined in Fig. 7a, b, respectively. To our surprise, the enriched KEGG terms of miR-106a and miR-106b were approximately the same. What’s more, we identified several pathways from the top 20 enriched KEGG terms, namely pathways in cancer, p53 signaling pathway, cell cycle, TGF-beta signaling pathway and Proteoglycans in cancer, which were related to the occurrence and development of GC.

The consistent enrichment results of miR-106a and miR-106b including GO and KEGG pathway analysis indicated that although the different location on chromosome, miR-106a and miR-106b may have the similar functions and could be both used in detecting GC. Meanwhile, the GO and KEGG pathway enrichment results explained why miR-106 why miR-106 could serve as a promising biomarker for detecting GC to some extent.

### PPI network construction and identification of key target nodes

**Fig. 8:**
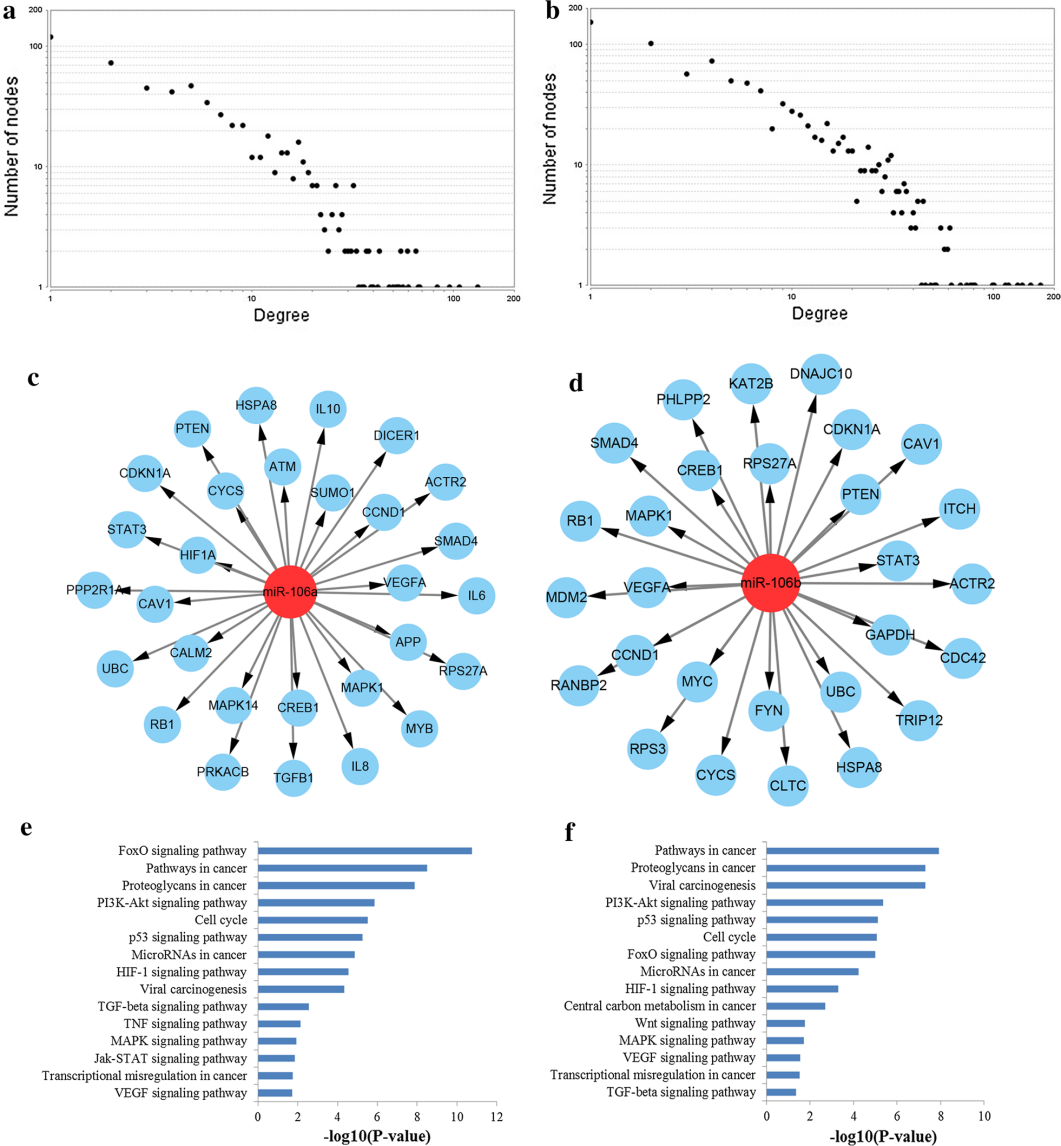
PPI network construction results. **a** Degree distributions of nodes for network constructed with miR-106a targets; **b** degree distributions of nodes for network set up with miR-106b targets; **c** hub genes of network for miR-106a targets; **d** hub genes of network for miR-106b targets; **e** pathway enrichment results for the selected hub genes of miR-106a targets network; **f** pathway enrichment results for the selected hub genes of miR-106b targets network. *PPI* protein–protein interaction

The information retrieved by STRING was integrated and set up the PPI network of miR-106a and miR-106b targets, respectively. As a result, a PPI network with statistical significance made up of 639 nodes was identified with the set of 896 target genes of miR-106a. Meanwhile, with the set of 1156 target genes of miR-106b, a statistically significant network consisting of 960 nodes were screened. We estimated three network parameters including degree centrality, betweenness centrality, and closeness centrality of the constructed network to explore the relationships between microRNA targets. Each method screened the hub genes in the network. Top 50 genes evaluated by the three methods in the PPI network were screened and intersected. Finally, 30 and 29 genes which could be screened by all the three methods were identified as hub genes for miR-106a and miR-106b, respectively. The regulatory networks were reconstructed with miR-106a and miR-106b and their identified hub nodes, plotted in Fig. 8. Furthermore, we evaluated the biological function of the selected key miR-106 targets, we found that these key target nodes regulated by miR-106a and miR-106b both played a role in FoxO signaling pathway, pathways in cancer, PI3K-Akt signaling pathway, cell cycle, and p53 signaling pathway (Fig. 8).

**Fig. 9:**
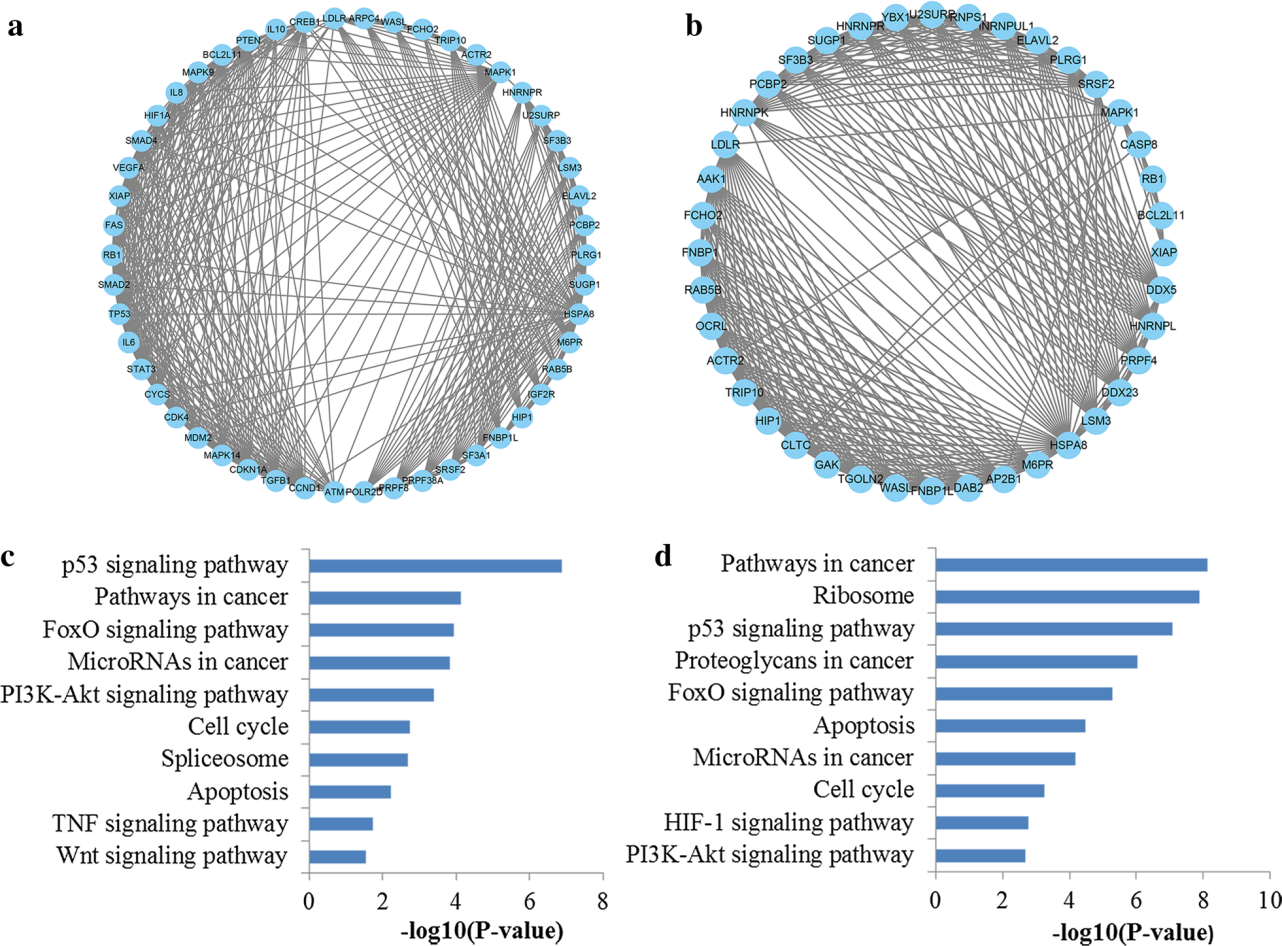
The significant modules from the PPI network. **a** The significant module in the PPI network for miR-106a targets; **b** the significant module in the PPI network for miR-106b targets; **c** pathways enriched by all the nodes involved in the identified module for miR-106a; **d** pathways enriched by all the nodes involved in the identified module for miR-106a. *PPI* protein–protein interaction

Next, active modules were identified (Fig. 9). According to KEGG pathway enrichment analysis, the genes involved in the significant module of miR-106a targets network were mainly associated with p53 signaling pathway, pathways in cancer, FoxO signaling pathway, microRNAs in cancer, PI3K-Akt signaling pathway and cell cycle while the genes involved in the significant module of miR-106b targets network were related to pathways in cancer, p53 signaling pathway, FoxO signaling pathway, microRNAs in cancer and cell cycle.

## Discussion

Over the decades, microRNAs have gained great attention in scientific researches for cancer detection, diagnosis, and treatment as they possess perfect biomarker characteristics and are highly involved in the occurrence and development of a variety of cancers [35]. As one of the most researched microRNAs, miR-106 has been suggested by emerging evidences that it could be a novel potential biomarker for GC detection. However, the detection accuracy was inconsistent among a series of quantitative analyses. These conflicting conclusions prompted us to employ this comprehensive and up-to-date research so that a conclusion on the diagnostic power of miR-106 for monitoring GC can be drawn. Meanwhile, we conducted an integrative functional analysis of miR-106 to understand the question why it could help distinguish GC patients from normal controls.

In the present study, we found that miR-106 achieved the overall pooled sensitivity of 0.71, specificity of 0.82, and AUC of 0.80, indicating a moderate overall accuracy. Circulating miR-106 as a more researched diagnostic marker in GC detection compared with other sample sources yielded a pooled sensitivity of 0.71, specificity of 0.82, and AUC of 0.81. Subgroup analysis indicated that sample type and sample size may influence the diagnostic accuracy. Specifically, it was revealed that plasma-based assays and miR-106 assays with a large sample size had significantly better overall diagnostic accuracy than serum-based ones and miR-106 assays with a small sample size, respectively. Meanwhile, although located in different chromosomes, miR-106a yielded a similar diagnostic accuracy compared with miR-106b.

Up to now, most attention on biomarker prediction in GC has been absorbed in single biomarkers. Actually, single biomarker is hard to reveal GC evolutionary process at the systems biology level as GC is a highly heterogeneous disease. On the contrary, combination markers may be more reliable with greater power for explaining the internal mechanisms of GC [36]. Therefore, we performed an analysis for miR-106-related combination markers in GC to investigate whether they were more powerful than miR-106 alone in detecting GC. It is worth noting that serum miR-106-related combination markers had a higher level of predictive power than serum miR-106 alone. However, it is difficult for us to conduct further investigations due to the limited number of studies enrolled in the analysis for miR-106-related combination markers.

We also performed integrative and comprehensive bioinformatics analysis to explore the function of miR-106 at the systems biology level. Most GO terms enriched by miR-106a and miR-106b target genes were both significantly associated with the regulation of transcription, apoptotic process at the BP level, basic cell structures at CC level along with the binding functions such as protein binding, DNA binding, transcription factor binding at MF level. Furthermore, the enriched KEGG pathways of miR-106a and miR-106b target genes were approximately the same, including pathways in cancer, p53 signaling pathway, cell cycle, TGF-beta signaling pathway and proteoglycans in cancer, which were highly associated with the occurrence and development of GC. Interestingly, although the different locations on chromosome, the targets of miR-106a and miR-106b fall into the similar functional modules, pathways or networks and then become more consistent when enriched to systems biology levels. In general, functionally concerned genes often emerge a coordinated expression to exert their roles in the same functional modules, indicating that miR-106a and miR-106b may have a synergistic effect in the initiation and progression of GC. The above results not only demonstrated the robustness of our study but explained why miR-106 could serve as a promising biomarker for detecting GC to some extent.

To further reveal the correlations among the target genes of miR-106a and miR-106b, we performed the PPI network analysis. Through PPI network construction, a series of hub genes were screened by three different network analysis methods. In our study, it was revealed that these key target nodes regulated by miR-106a and miR-106b both participated in FoxO signaling pathway, pathways in cancer, PI3K-Akt signaling pathway, cell cycle, and p53 signaling pathway. What’s more, module analysis of the PPI network revealed that the most significant modules of miR-106a and miR-106b targets network were both associated with p53 signaling pathway, pathways in cancer, FoxO signaling pathway, microRNAs in cancer, PI3K-Akt signaling pathway and cell cycle. PPI network analysis including hub genes identification and module analysis revealed the function of miR-106 again.

Based on above results, we found that several pathways were repeatedly mentioned in KEGG pathway analysis enriched by all the miR-106a and miR-106b targets, key hub targets and network modules, including p53 signaling pathway, pathways in cancer, FoxO signaling pathway, PI3 K-Akt signaling pathway and cell cycle. Pathways in cancer consist of several well-known signaling pathways including TGF-β, MAPK, Wnt and p53, which play important roles in cell apoptosis, proliferation, differentiation, invasion and metastasis. The well-studied p53 pathway, perhaps the most vital determinant of carcinogenesis, has been inextricably linked to establishment and progression of almost all types of cancer including GC [37, 38]. Cell cycle, another very important signaling pathway, contributes to the malignant progression of various human cancers including GC due to the involvement in cell growth, differentiation and apoptosis, as well as cancer development and metastasis [39]. Recent studies have proposed that the activation of the PI3K/Akt pathway may be responsible for the tumorigenesis by playing a pivotal role in control of cell cycle and survival of cell [40]. The information gathered so far indicates that FoxO signaling pathway could play vital roles in mediating apoptosis and thus determines cell death and survival [41]. In short, all the above pathways have been verified by the published literatures involved in the tumorigenesis and progression of GC, which may provide new ideas for the molecular mechanisms of miR-106 in GC.

Although mounting evidence from diagnostic tests indicated miR-106 as a promising GC marker, difficulty still remains for its application to clinical practice. There are several points we can do to optimize the miR-106 assay. Firstly, an appropriate standard cut-off value, consistent detection and normalization methods for miR-106 expression are required. Secondly, it was revealed from our results that plasma miR-106 may be a more powerful marker for detecting GC compared with serum miR-106. So plasma could be selected as the suitable sample source for further detection. Thirdly, as indicated in our study, sample size influenced the sensitivity and specificity. Larger sample size exhibited higher diagnostic accuracy. Thus, further large-scale prospective studies are warranted to develop integrative diagnostic models with more appropriate and better prediction capacity. Fourthly, although miR-106a and miR-106b are members of different paralogous clusters and located on different chromosomes, there has been some evidence in the literature, that both these two microRNAs can co-expressed in gastric tumor tissues. Based on our results, both miR-106a and miR-106b could be evaluated in diagnostic samples for diagnostic purposes of gastric cancer. In addition, combination biomarkers, which are combinations of several markers, have been shown to improve the prediction accuracy compared with single biomarker. According to our findings, single miR-106 was significant but not strong enough to undertake early diagnosis, while miR-106-related combination markers improved the diagnostic accuracy. The combination of miR-106 and other microRNAs may be the right way to solve the limited accuracy. Moreover, it has been reported that combination of protein-biomarkers and microRNAs may be an effective way to improve the diagnostic accuracy [42]. So more attempts are required for evaluating the combination biomarkers in the further study.

Our study had several important strengths. First, we carried out a relatively thorough systematic search and applied a comprehensive analytic approach to investigate the diagnostic power of miR-106 in patients with GC. Next, we evaluated the diagnostic value of miR-106-related combination markers in GC for the first time. It was suggested that the combination of miR-106 with other microRNAs improved the diagnostic accuracy, which may provide a novel potential tool for progress in a clinical context. Moreover, we performed integrative and comprehensive bioinformatics analysis to explore the function of miR-106 at the systems biology level, explaining the reason why miR-106 could be used in the diagnosis of GC. However, the power of our study was limited by a few factors. Firstly, most studies in the diagnostic tests enrolled healthy participants as controls and were not blind in design, which limits the diagnostic performance. Secondly, some key information including stage of cancer, sex proportions and age distributions was not known, so further analysis could not be carried out. Thirdly, there were no studies investigating the non-Asian population, which may cause potential heterogeneity from ethnicity. Fourthly, the sources of sample were inconsistent including plasma (n = 5), serum (n = 5), gastric juice (n = 1) and tissue (n = 1). Accordingly, subgroup analysis by specimen could not be performed for the limited individual sample size. In addition, the number of studies and sample sizes enrolled in the analysis for miR-106-related combination markers are limited, which make it difficult for us to conduct further investigations. As the miR-106 combination markers are all different in all six studies, it still remains an open question which should be combined with miR-106 for improving the diagnostic power.

## Conclusion

Taken together, in this study, it is concluded that miR-106 is a useful biomarker for GC detection. Prospectively, combining miR-106 and other microRNAs may be considered as more powerful diagnostic tools for clinical application than individual miR-106. Integrative and comprehensive bioinformatics analysis was performed to explore the function of miR-106 at the systems biology level. Nonetheless, further large-scale prospective studies are needed to create integrative diagnostic models with more pronounced accuracy.

## Abbreviations

GC: gastric cancer
AUC: area under the curve
GO: gene ontology
KEGG: Kyoto Encyclopedia of Genes and Genomes
PPI: protein–protein interaction
QUADAS-2: Quality Assessment of Diagnostic Accuracy Studies 2
TP: true positive
FP: false positive
FN: false negative
TN: true negative
PLR: positive likelihood ratios
NLR: negative likelihood ratios
DOR: diagnostic odds ratio
CI: confidence interval
SROC: summary receiver operator characteristic
DAVID: Database for Annotation, Visualization, and Integrated Discovery
STRING: Search Tool for the Retrieval of Interacting Genes
MCODE: Molecular Complex Detection
BP: biological process
CC: cellular component
MF: molecular function
NA: not available
qPCR: quantitative real-time PCR
